# Synthesis of silver nanoparticles embedded with single-walled carbon nanotubes for printable elastic electrodes and sensors with high stability

**DOI:** 10.1038/s41598-021-84386-4

**Published:** 2021-03-04

**Authors:** Jae-Won Lee, Joon Young Cho, Mi Jeong Kim, Jung Hoon Kim, Jong Hwan Park, Seung Yol Jeong, Seon Hee Seo, Geon-Woong Lee, Hee Jin Jeong, Joong Tark Han

**Affiliations:** 1 Nano Hybrid Technology Research Center, Creative and Fundamental Research Division, Korea Electrotechnology Research Institute (KERI), Changwon, 51543 South Korea; 2 Department of Physics, Pusan National University, Busan, 46241 South Korea; 3 Department of Electro-Functionality Material Engineering, University of Science and Technology (UST), Changwon, 51543 South Korea; 4 Department of Chemical Engineering, Pohang University of Science and Technology, Pohang, 37666 South Korea

**Keywords:** Materials science, Nanoscience and technology

## Abstract

Soft electronic devices that are bendable and stretchable require stretchable electric or electronic components. Nanostructured conducting materials or soft conducting polymers are one of the most promising fillers to achieve high performance and durability. Here, we report silver nanoparticles (AgNPs) embedded with single-walled carbon nanotubes (SWCNTs) synthesized in aqueous solutions at room temperature, using NaBH_4_ as a reducing agent in the presence of highly oxidized SWCNTs as efficient nucleation agents. Elastic composite films composed of the AgNPs-embedded SWCNTs, Ag flake, and polydimethylsiloxane are irradiated with radiation from a Xenon flash lamp within a time interval of one second for efficient sintering of conductive fillers. Under high irradiation energy, the stretchable electrodes are created with a maximum conductivity of 4,907 S cm^−1^ and a highly stretchable stability of over 10,000 cycles under a 20% strain. Moreover, under a low irradiation energy, strain sensors with a gauge factor of 76 under a 20% strain and 5.4 under a 5% strain are fabricated. For practical demonstration, the fabricated stretchable electrode and strain sensor are attached to a human finger for detecting the motions of the finger.

## Introduction

Soft electronics technology has advocated for the use of stretchable interconnecting electrodes or sensors under various harsh mechanical deformations because of their potential applications in healthcare, sports performance monitoring, personalized medical rehabilitation, and soft robotics, etc^1,2^. Conventionally, metal nanowires, metal flakes and conducting polymers have been used as conductive fillers to fabricate high-performance stretchable electrodes or strain sensors^3–15^. Moreover, in the case of metal/nanocarbon composite materials, carbon nanotubes (CNTs) or graphene nanosheets were decorated with metal nanoparticles. Decoration of a CNT surface with metal nanoparticles or metal belts has been implemented by attaching organic moieties on that surface, which can interact with metal ions, activating nucleation of metal nanoparticles^4,16–25^. However, the most reliable composite structure is that the part of mechanically stable nanocarbon materials having high electrical conductivity are incorporated inside metal particles. Then under harsh mechanical deformation, conducting fillers in the elastic polymer matrix can be interconnected with each other efficiently and with high durability. Until now, a bottom-up approach method for the synthesis of CNT embedded metal particles had not been reported. This was because of the lack of an efficient oxidation method without the detrimental cutting for CNT surfaces that have a high content of oxidative functional groups. Recently, it was reported that the surface of a vulnerable single-walled CNTs (SWCNTs) can be highly oxidized by using a small amount of acid and a strong oxidant there by mimicking the flour dough kneading process^26^. Highly oxidized SWCNTs (Ox-SWCNTs), having many oxidative functional groups such as the carboxyl, hydroxyl, carbonyl, etc. can be a good nucleation template for metal nanoparticles.

Here, we report that SWCNT-embedded silver nanoparticles were synthesized at room temperature by using, Ox-SWCNTs, a silver precursor and a reducing agent in an aqueous solution. The silver particle size was controlled by varying the mass ratio of SWCNTs and Ag precursor ratio from 50 nm to several micrometres. Moreover, more uniformly sized Ag nanoparticles were synthesized by using a Couette–Taylor flow reactor. The elastic composite films were then fabricated by screen-printing with a conducting paste that was prepared from the direct mixing of an elastomer, synthesized conducting materials and a solvent. To achieve suitability for applications in stretchable electrodes and strain sensors, the electrical conductivity, mechanical stability, and sensitivity of the composite films were rationally controlled by varying the conducting filler contents and the radiation energy from a Xenon flash lamp.

## Methods

### Materials

Single-walled carbon nanotube (SWCNT) powder (75% purity) synthesized using the chemical vapour deposition method was obtained from OCSiAl, Luxembourg. Fuming nitric acid was purchased from Tokyo Chemical Industry, and NaClO_3_, HCl and H_2_O_2_ were purchased from Samchun Chemicals. AgNO_3_ powder was obtained from Daejung. NaBH_4_ powder was obtained from Sigma-Aldrich and used without any further treatment for the oxidation and synthesis of SWCNT and ox-SWCNT/Ag NP. To synthesize the elastic composite paste, Ag flake and PDMS (Sylgard 184) were purchased from Chang Sung Corp. and Dow Corning, respectively.

### Synthesis of oxidized SWCNTs and ox-SWCNT dispersion

The oxidation of SWCNTs was carried out using the kneading process. Before addition of a strong acid solution, 1 g of SWCNT powder and 7.5 g of sodium chlorate (NaClO_3_) powder were mixed with a high speed mixer in order to distribute tiny NaClO_3_ particles between the SWCNT networks. This promotes the dissolution of NaClO_3_ in the acid resulting in an efficient oxidation of the SWCNTs. Then, 20 mL of fuming HNO_3_ was poured slowly into the mixed powder while kneading with a Teflon spatula for a few minutes. The mixture was kept at room temperature for 1 h; 1 L of deionized water was then added to the mixture in order to dilute the acid. Subsequently, HCl and H_2_O_2_ were added to remove metal ions and terminate the reaction. The resulting mixture was centrifuged to remove residual acid and oxidant molecules, resulting in an Ox-SWCNT paste. To fabricate the Ox-SWCNT dispersion in DI water with 500 mg L^−1^, the horn sonicator was used without any dispersant molecules for 5 min.

### Synthesis of the SWCNT-embedded AgNPs in the flask

The Ox-SWCNT dispersion was poured while stirring into a 3-neck round bottom flask and stirred at room temperature. A 0.08 M AgNO_3_ solution was prepared using DI water and stirred for 10 min at room temperature to dissolve the AgNO_3_ powder. After stirring the AgNO_3_ solution was injected into the SWCNT dispersion and stirred for 30 min. In addition, 0.6 M NaBH_4_ solution was injected into the dispersion using a feeding pump at a rate of 1 mL min^−1^. After the NaBH_4_ solution had been totally injected, the solution was stirred overnight. To terminate the reaction, the mixture was washed with DI water using centrifugation.

### Synthesis of SWCNT-embedded AgNPs in the Couette–Taylor reactor

The Ox-SWCNT dispersion (500 mg L^−1^) was put in a Couette–Taylor reactor and the inner cylinder rotation rate was set as 1000 rpm. A 0.2 M aqueous AgNO_3_ solution was injected into the Couette–Taylor reactor and mixed with the SWCNT dispersion. After 10 min of mixing, the previously prepared 0.6 M NaBH_4_ solution was injected into the reactor with a feeding pump at various rates. After injecting all the NaBH_4_ solution, the reaction continued for a further 6 h. The resultant Ox-SWCNT/Ag hybrid materials were washed with DI water using centrifugation. This whole process was performed under ambient conditions.

### Synthesis of elastic composite film

Composite pastes were prepared by mixing the commercial Ag flake (2 µm, Chang Sung Corp.), SWCNT-embedded AgNPs, and PDMS. The contents of the conducting filler with respect to the PDMS elastomer (Sylgard 184, Dow Corning Corp.) was varied from 15 to 23 vol% in order to optimize the performance of the stretchable electrodes and sensors. The weight ratio of Ag flake and SWCNT-embedded AgNPs was fixed at 1:0.6. Conducting fillers and as-prepared PDMS (mixing a PDMS rubber and surfactant with a weight ratio of 10:1) were mixed with a planetary mixer (ARE-310, Thinky) for 0.5 h at 2000 rpm. Prepared composite pastes were screen-printed on a PDMS substrate using a patterned screen mask (SUS 325 mesh) with an emulsion mask thickness of 10 µm, then followed by drying at 100 ℃ for 1 h. The embedding structure of SWCNT in AgNPs did not damaged during this mechanical fabrication process of stretchable paste as shown in Figure S7. IPL sintering of the elastic composite films was carried out using a Xenon flash lamp with a broad wavelength range from 300 to 900 nm. Films were irradiated with IPL energy density between 10 to 20 J cm^−2^. All methods and experiments were carried out in accordance with the relevant guidelines and regulations. Informed consent was obtained from the participation in the experiment.

### Characterization

The surface morphology of Ox-SWCNTs and AgNPs was observed using an atomic force microscope (AFM), (Nanoscope Multimode system, Veeco Instruments) and a field-emission scanning electron microscopy (FE-SEM, Hitachi S4800). A high-resolution transmission electron microscope (HR-TEM, Titan G2 60-300, FEI) was used to observe the formation of AgNPs on the surface of Ox-SWCNT bundles at an accelerating voltage of 80 kV. The chemical structure of Ox-SWCNTs was characterized using an X-ray photoelectron spectroscopy (XPS, K-alpha + system, Thermo Fisher Scientific) with mono-chromated Al K α X-ray radiation as the excitation source. The crystalline structure of SWCNT-embedded AgNPs and AgNPs was characterized using an X-ray diffraction (XRD, Philips PW 3830) with Cu, Kα radiation (λ = 1.5418 Å). The structural characteristics of Ox-SWCNTs and SWCNT-embedded AgNPs were identified using Raman spectrometry (NTEGRA SPECTRA, NT-MDT) with an excitation wavelength of 633 nm. The electrical conductivity of the elastic conducting films was measured using a four-point probe method (Loresta, MCP-T610). The electrical conductivity and resistance change for the stretchable electrodes and sensors were measured using a Keithely 2636B source meter connected to a custom-built automatic stretching tester. Before using humans as test subjects, informed consent from the human subjects was obtained appropriately in accordance with the guidelines of the research ethics in science and engineering approved by Korean Federation of Science and Technology Societies.

## Results

**Figure 1:**
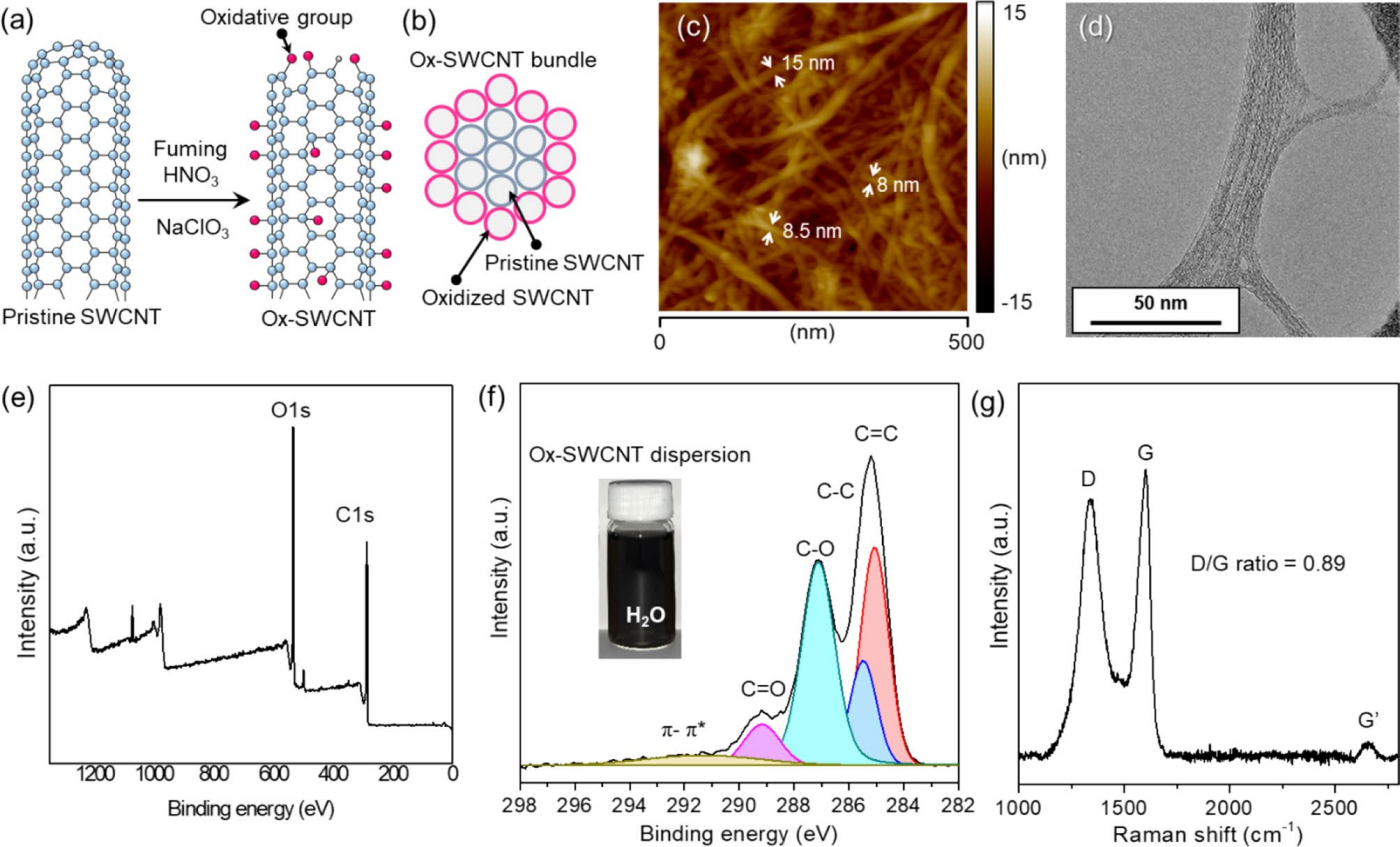
Illustration of (**a**) fabrication of Ox-SWCNTs with using fuming HNO_3_ and NaClO_3_ (**b**) the cross-sectional structure of the Ox-SWCNT bundle; core SWCNTs remain intact structures. Characteristics of Ox-SWCNTs: (**c**) AFM image, (**d**) TEM image, (**e**) survey scan plot in XPS, (**f**) C1s plot in XPS, (**g**) Raman spectrum.

To enhance the affinity between the Ag precursor and SWCNTs for the nucleation of AgNPs, the SWCNT surface must be highly oxidized thus maintaining the SWCNT dimension. Harsh oxidation conditions from using a strong acid and oxidant cause detrimental effects on the CNT structures resulting in unzipping and cutting^27,28^. Particularly, SWCNTs are vulnerable to be cut in harsh oxidation conditions because of the pyramidalization effect of sp^2^ hybridized carbon atoms, which enhances the chemical reactivity of the side wall of the SWCNT. In this study, to counteract this concern, the SWCNTs were efficiently oxidized by using a chlorate-based oxidation method and applying the kneading process as previously reported^25^ (Fig. 1a). As illustrated in Fig. 1b, pristine SWCNTs in the core still have their intrinsic structures and the outside SWCNTs are highly oxidized, makes them a nucleation site of AgNPs. Essentially, to enhance the oxidation efficiency, 1 g of the SWCNT and 10 g of NaClO_3_ powder were mixed and grinded by a high speed blade mixer, prior to adding 50 mL of fuming HNO_3_ for kneading (Figure S1). The SWCNT/NaClO_3_/fuming HNO_3_ mixture was then kneaded using a Teflon spatula for 10 min and finally kept at room temperature for 1 h. Reducing the particle size of NaClO_3_ powder increases its solubility in fuming HNO_3_ and results in uniformly distributed SWCNT networks, which contributes to a fast oxidation process, as quick as even taking 1 h to complete. The resultant Ox-SWCNT bundles have an average bundle size of 10 nm and a bundle length of more than 5 μm (Fig. 1c). The TEM image in Fig. 1d shows that the surfaces of the SWCNT bundles have limited crystalline structures because of the introduction of oxidative groups. The pristine SWCNTs exist at the core of the bundle as illustrated in Fig. 1b. The XPS spectrum in Fig. 1e shows that the SWCNTs were highly oxidized and consist of a C/O atomic ratio of 2.34; deconvolution of C1s peak shows that the SWCNTs were functionalized with mainly hydroxyl and carboxyl groups (Fig. 1f). This functionalization led to an increase in the D band (D/G intensity ratio = 0.89) of the Raman spectrum (Fig. 1g). The number of functional groups and the extent of the D band intensity can be controlled by varying the ratio of NaClO_3_: SWCNTs or by varying the duration of the oxidation process. The Ox-SWCNTs were easily dispersed in water by using a homogenizer without dispersant to synthesize AgNPs.

**Figure 2:**
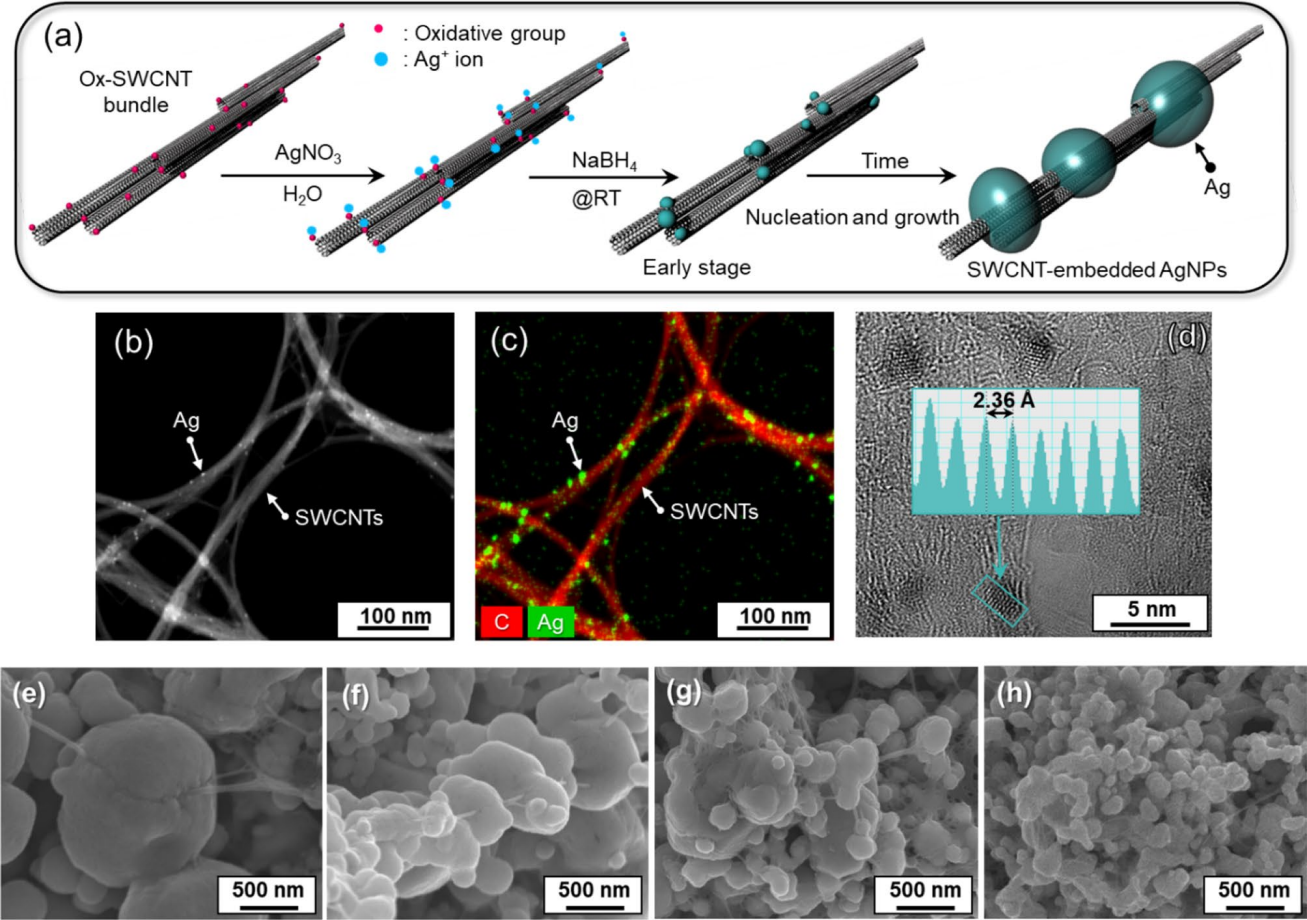
(**a**) Schematics of synthesis of SWCNT-embedded AgNPs. (**b**) TEM image analysis of SWCNTs decorated with tiny Ag particles nucleated by oxidative functional groups at early stage: (**c**) EDS mapping image of Ag and carbon, (**d**) High resolution TEM image and d-spacing line profile of Ag nuclei crystal (inset). FESEM images of SWCNT-embedded AgNPs by varying the concentration of AgNO_3_ in the solution; (**e**) 0.08 M, (**f**) 0.06 M, (**g**) 0.04 M, (**h**) 0.02 M.

The morphology of AgNPs can be affected by many parameters, such as type of reducing agent and its solvent, concentration, the feeding rate of reducing agents, temperature, and additives such as stabilizers or complexing agents. In this study, as shown in Fig. 2a, an aqueous solution of NaBH_4_ was used as the reducing agent for the Ag nanostructure formation and Ox-SWCNTs were utilized as a nucleation template. Before feeding NaBH_4_ to the aqueous Ox-SWCNT dispersion, 12.5 mL of AgNO_3_ solution (0.08 M) was mixed with 62.5 ml of Ox-SWCNT dispersion (500 mg L^−1^) and then stirred for 30 min to enhance the interaction between the Ag^+^ ions and the functional groups on the Ox-SWCNT surface, as shown in Fig. 2a. In the presence of Ox-SWCNTs, microscopic AgNPs can be nucleated on the surface. To verify the occurrence of this process, after 10 s of feeding NaBH_4_ (0.48 M), the nanostructure of Ox-SWCNTs were characterized using high resolution TEM. As shown in Fig. 2b, numerous minuscule particles 2.5–3 nm were decorated on the Ox-SWCNT surface, which were identified as AgNPs from an EDAX analysis (Fig. 2c). The high resolution TEM image in Fig. 2d shows the typical d-spacing of the Ag (111) crystal measuring 2.36 Å. This result indicates that SWCNTs can be decorated with metal nanoparticles without modifying the SWCNT surface with ionic organic materials.

**Figure 3:**
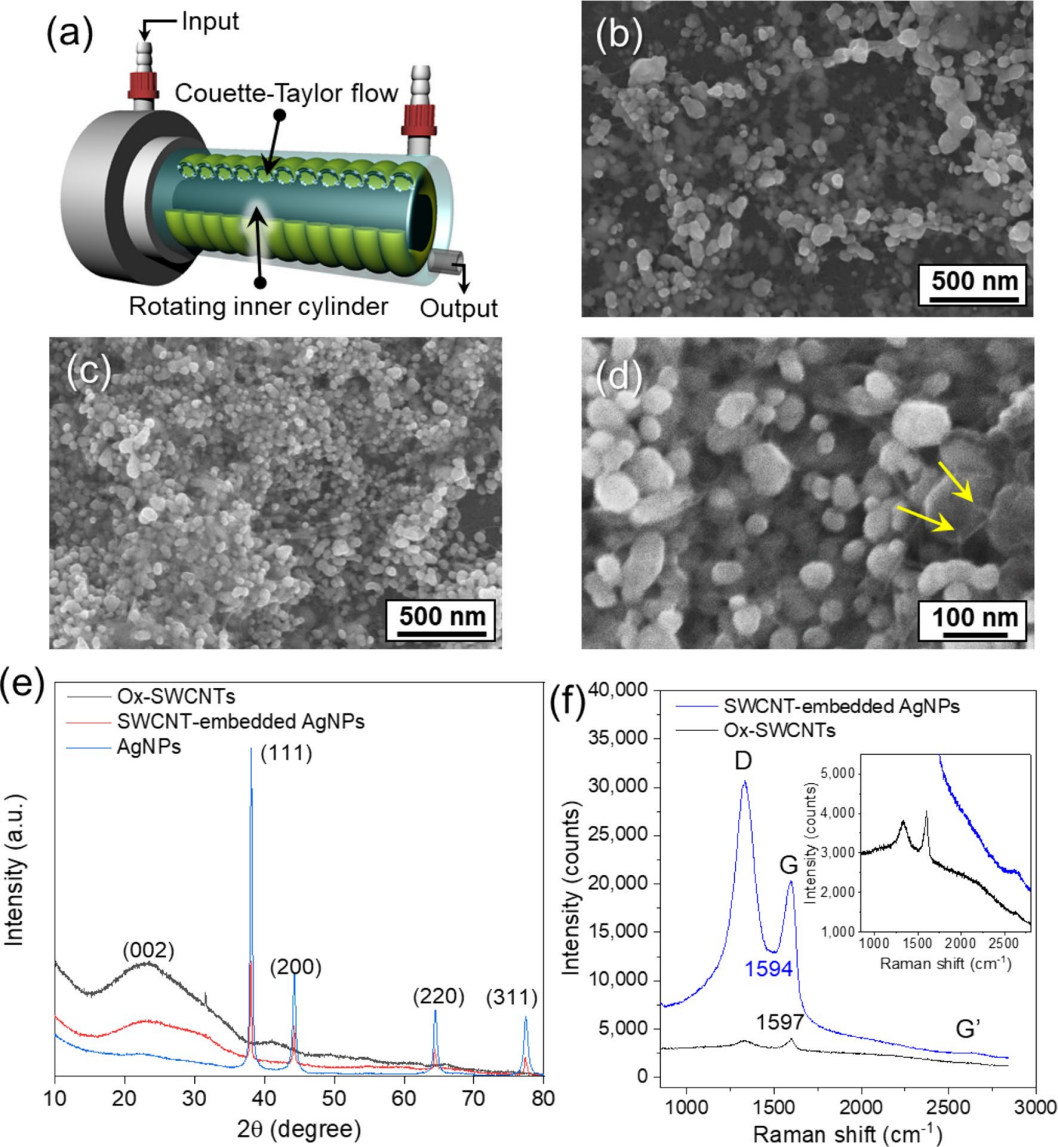
(**a**) Illustration of the Couette–Taylor reactor. FESEM image of SWCNT-embedded AgNPs in the C–T reactor by varying the feeding rate of the reducing agent, NaBH_4_; (**b**) 5 ml min^−1^, (**c**) 50 ml min^−1^. (**d**) Magnified FESEM image of (**c**). (**e**) XRD pattern of SWCNT-embedded AgNPs. (**f**) Raman spectra of Ox-SWCNTs and SWCNT-embedded AgNPs by 5 s data accumulation. Inset in (**f**) is a magnified Raman spectrum of Ox-SWCNTs with a weak peak intensity.

Since the concentration of AgNO_3_ and Ox-SWCNTs in the solution is vital in controlling their structure, the crystallization of Ag in the presence of Ox-SWCNT was investigated by varying the concentration of AgNO_3_ in different solutions. Figure 2e–h presents representative morphologies of the Ag nanostructures obtained from different AgNO_3_ solutions with concentrations ranging from 80 to 20 mM. It is important to note that Ox-SWCNTs were embedded in the synthesized AgNPs like a pearl necklace, and the size of AgNPs gradually decreased from values above 500 nm to values below 100 nm. During synthesis of AgNPs, their shape can be controlled by using the stirring method, which influences the nucleation rate. To control the shear forces during the reduction of Ag^+^ ions in the presence of Ox-SWCNTs, a Couette–Taylor (C–T) reactor was utilized. The C–T reactor has been widely used to synthesize nanoparticles owing to the C–T flow forming in the gap between two rotating cylinders, as presented in Fig. 3a. As expected, using the C–T flow reactor, the smaller AgNPs incorporated with the SWCNTs that were synthesized (Fig. 3b). Furthermore, a faster feed rate of the reducing agent generated smaller AgNPs (Fig. 3c,d). Notably, the Ox-SWCNTs were still embedded onto the AgNPs, which means that the oxidative functional groups on the SWCNTs work as nucleation sites even under high shear flow conditions. The X-ray diffraction (XRD) pattern of these SWCNT-embedded AgNPs demonstrates the co-existence of SWCNTs and AgNPs (Fig. 3e). The SWCNT bundle produced a broad peak at 26.2°, which corresponds to a typical intertube spacing of 3.4 Å. Several diffraction peaks corresponding to the crystalline planes of (111), (200), (220), (311) and (222) of metallic Ag with face-centred cubic (fcc) structure were detected at 38.12°, 44.19°, 64.65°, 77.41°, and 81.43° (JCPDS No. 04-0783), respectively^29^.

To elucidate the crystalline structure of Ox-SWCNTs after synthesis of AgNPs, Raman spectroscopy was employed using laser at a wavelength of 633 nm; the laser exposure time was 5 s. The Raman spectra of pristine- and Ox-SWCNTs was compared with that of the SWCNT-embedded AgNPs as shown in Fig. 3f. The intensity of Raman scattering of pristine SWCNTs was depressed after oxidation due to the reduction of the resonance enhancement effect of the Ox-SWCNTs^30^. However, for the SWCNT-embedded AgNPs, the Raman scattering intensities for the D and G band of the SWCNTs are drastically amplified compared with that of the Ox-SWCNTs. This means that the synthesized SWCNT-embedded AgNPs can play as a surface enhanced Raman scattering (SERS) substrate. On the nanostructured Ag surface, laser excitation of Ag nanostructures, resonantly drives the surface charges, creating a highly localized plasmonic light field. Notably, the typical disorder-induced D band at 1343 cm^−1^ is more enhanced than the G band at 1597 cm^−1^ for the SWCNT-embedded AgNPs, and the G band shifted to 1594 cm^−1^ by dedoping with Ag. D/G intensity ratios of Ox-SWCNTs and SWCNT-embedded AgNPs were 0.89 and 1.3, respectively. The D band of the SWCNT is the double resonance intervalley process of elastic scattering caused by a defect of the SWCNT, while the G band originates from the first order resonance Raman scattering process^30^. This result indicates that the double resonance Raman scattering is more sensitive to the SERS substrate than the first order Raman process. Moreover, this result indicates that during the chemical reduction of Ag^+^ ions by NaBH_4_, Ox-SWCNTs were not chemically reduced, implying that most of the oxidative functional groups interact with Ag^+^ ions before chemical reduction. Without Ag^+^ ions, the Ox-SWCNTs were reduced by deoxygenation using NaBH_4_ at room temperature (Figure S2).

**Figure 4:**
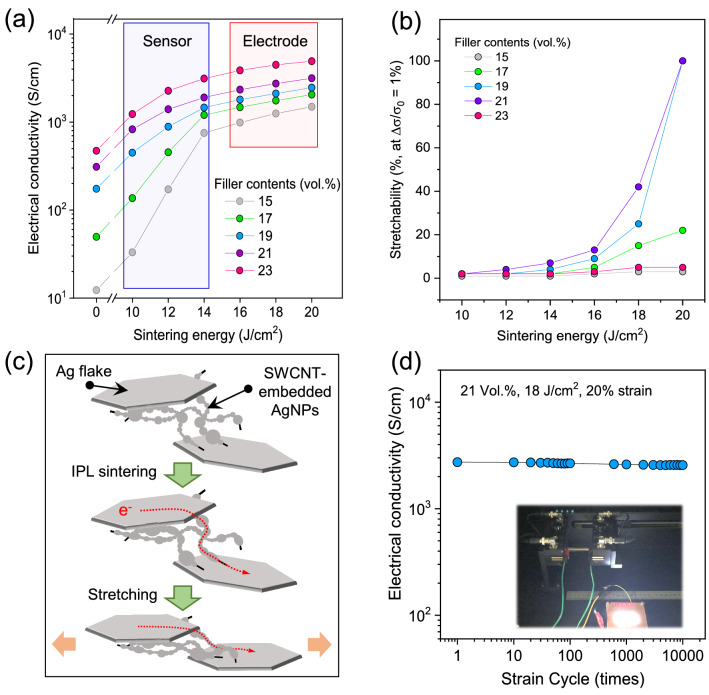
(**a**) Electrical conductivity and (**b**) stretchability at 1% of relative electrical conductivity change for the composite films with different filler contents ranging from 15 to 23 vol% sintered by various IPL energies. (**c**) Schematic image of conduction mechanism for the composite films composed of the Ag flake, SWCNT-embedded AgNPs, and PDMS pre-polymer during stretching. (**d**) Repeatability test of up to 10,000 cycles for the composite film under 20% mechanical strain.

In order to investigate the electrical properties under mechanical deformation conditions, composite pastes comprising Ag flakes, SWCNT-embedded AgNPs, and polydimethylsiloxane (PDMS) pre-polymer were screen-printed on the PDMS substrate. Ag flakes have been solely used as a stretchable conducting filler due to their large contact area^31^; their hybridization with AgNPs or carbon nanotubes could enhance electrical and stretchable properties^4,14^. Figure 4a shows intrinsic electrical conductivities of the composite films with varying conducting filler contents, without any mechanical strains. As expected, the electrical conductivity gradually increased as the filler contents increased, reaching a conductivity of 472 S cm^−1^ at 23 vol%, which is relatively low compared to previously reported literature^4,14^. This is probably because of the limited electrical contact area between the conducting fillers. As shown in Fig. 2b–d of SEM images for the as-synthesized SWCNT-embedded AgNPs, most of the AgNPs are spherical shaped and connected through point-to-point contact. The electrical failure was observed at high filler contents of more than 23 vol% because of the cracks or tears in the composite films. To increase the contact area and thus enhance the electrical conductivity of the composite films, IPL sintering using a Xenon flash lamp with a broad wavelength spectrum was employed. IPL-induced heating of conductive nanomaterials has recently attracted a lot of interest in the field of flexible and stretchable applications, because it allows for the ultrafast heating of nanomaterials without damaging or deforming polymer substrates^32,33^. Conductive nanomaterials can play a significant role as an efficient and immediate source of heat, because the heating is considerably related to the localized surface plasmon resonance effect which resulted from the coupling of surface plasmons of nanomaterials with light. Thus, when the filler contents and sintering energy were increased, the electrical conductivity also increased, reaching a maximum value of 4907 S cm^−1^ at 20 J cm^−2^ for a 23 vol% sample, which is 10.4 times greater than its initial value shown in Fig. 4a. High sintering energy of more than 20 J cm^−2^ gave rise to extremely high thermal energy in the sample, resulting in the severe damage of the PDMS elastomer.

To investigate the stretchable properties of the composite films, the electrical conductivity was measured under a mechanical strain ranging from 0 to 100%. Figure 4b shows the stretchability at 1% change of the electrical conductivity with respect to the filler contents and sintering energy. In the case of low filler contents (15 and 17 vol%) and sintering energy (10–16 J cm^−2^), the stretchability was less than 20%. This low stretchability could be attributed to the relatively low conducting path upon strain which in turn below percolation threshold. However, high stretchabilities of more than 20% were measured at sintering energies of more than 18 J cm^−2^ in the case of filler contents (19 and 21) vol%. This could be confirmed with well-distributed and highly dense conducting fillers over entire composite films as shown in Figure S3. SWCNT-embedded AgNPs might play a key role for this high stretchability, as shown in the scheme of Fig. 4c. During the IPL sintering process, AgNPs synthesized on SWCNTs were connected to the Ag flakes as well as the neighbouring NPs; this resulted in the formation of an electron-transfer path. When mechanical strain is applied to the sample, the SWCNTs, as the efficient templates for the sintered NPs, can preserve the electron path due to their high durability upon stretching. A repeatability test (20% stretching) of up to 10,000 cycles for the composite film of 21 vol% filler contents and 18 J cm^−2^ IPL sintering shows a negligible change in the electrical conductivity (Fig. 4d), which is superior to previously reported results of Ag flake/AgNPs/PDMS and Ag/MWCNT/PDMS^4,14^. Similar results were observed for the composite film applied at higher mechanical strains (Figure S4). This high durable stretchability was confirmed by the absence of flickering or brightness decrease in the LED light connected with the composite film during the stretching test, as shown on the inset of Fig. 4d and Video S1. For a comparison, we carried out the repeatability test for the composite films composed individually of Ag flake and PDMS as well as Ag flake, AgNPs, SWCNT and PDMS. Even though the initial electrical properties were very similar to that of the AgNPs-embedded SWCNT, after IPL sintering, the resistivity was gradually increased as the number cycles increased; electrical failure was observed at 80 and 1,000 cycles, respectively, as shown in Figure S5a and Figure S5b. The SWCNTs could not hold and support the sintered AgNPs, so that the cracks were formed on the surface of the composite film upon stretching as shown on the inset of Figure S5b and Figure S5c.

**Figure 5:**
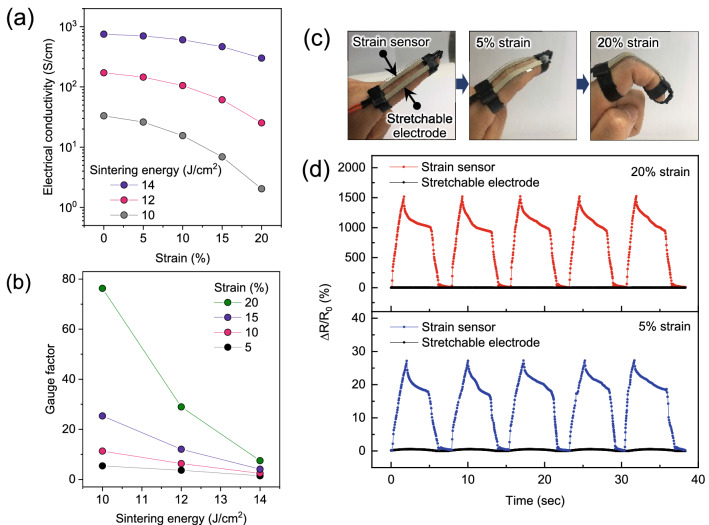
(**a**) Electrical conductivity and (**b**) calculated gauge factor for the composite films with respect to the sintering energy and strain. (**c**) Photographs of the stretchable electrode and strain sensor fabricated with the same PDMS substrate subjected with 5% and 20% strain and (**d**) their relative resistivity change profile for 5 times repeating tests.

In addition to its application in a stretchable electrode, the SWCNT-embedded AgNPs can also be used as an active material for a strain sensor. Figure 5a shows the electromechanical properties of the 15 vol% composite film sintered at various IPL energies ranging from 10 to 14 J cm^−2^. In the case of the composite film sintered at 14 J cm^−2^, the conductivity-strain slope was relatively small, even though the initial electrical conductivity was higher than for other sintering energies. Therefore, the gauge factor (GF) values were measured and found to be below 10, as shown in Fig. 5b. It is known that the sensitivity of a strain sensor can be evaluated using the GF; the equation to obtain GF values is shown below.

1 $${\text{GF}} = (\Delta R/R_{0} )/\varepsilon$$

*∆R*/*R*_0_ is the normalized change in the electrical resistance, and *ε* is the mechanical strain. In contrast, the electrical conductivity of the composite film sintered at 10 J cm^−2^ was significantly decreased when subjected to mechanical strain. The GF value was calculated to be 76 for a mechanical strain of 20%; this value is much higher than the conductor/elastomer-based stretchable strain sensors reported in previous literature^33–35^. This high sensitivity could be attributed to the embedded structure of the SWCNT inside AgNPs, which provides a long-range conductive path without suffering from the high contact resistance between junctions of the AgNPs.

For the feasibility of the stretchable devices in human motion detection, we fabricated the stretchable electrode and strain sensor in the same PDMS substrate as discussed earlier and attached it to a human finger as shown in Fig. 5c. Stretchable electrodes and strain sensors were prepared using 18 and 10 J cm^−2^ IPL sintering of a composite film with 21 and 15 vol% filler contents, respectively. Both ends were connected to conventional copper wires in order to measure the relative resistance change. Figure 5d shows the relative resistance change of the stretchable electrode and strain sensor when the finger is bent with a strain of 5% and 20%. The resistivity of strain sensor was significantly increased when the bending increased, but the stretchable electrode did not change as expected. The reproducible resistivity change for 5-time bending and releasing cycles indicated that the composite film could be used as a strain sensor with high mechanical stability. This behaviour was also confirmed by long-term durability results up to 500 cycles (Figure S6) and the flickering of the LED lights connected to the strain sensor in accordance with finger gestures (Video S2).

## Conclusion

CNT-embedded metal nanostructures are promising for realization of highly stable and stretchable electrode for soft electronics. In this study, silver nanostructures were systematically modulated by using SWCNTs that were highly oxidized by chlorate-based oxidation. It was demonstrated that this approach allows SWCNT-embedded Ag nanostructures to be synthesized in the presence of Ox-SWCNTs in environmentally friendly aqueous solutions at low temperature without any other additives. The SWCNT-embedded AgNPs as an efficient conductive filler were subsequently used to create highly conductive (4907 S cm^−1^) and highly stable and stretchable PDMS composite electrodes at over 10,000 cycles under a 20% strain. Strain sensors with a gauge factor of 76 under a 20% strain and 5.4 under a 5% strain are also achieved by rational irradiation of a Xenon flash lamp. It is hoped this approach will create new opportunities for development of conformable electrodes and sensors in soft electronics or wearable electronics.

## Supplementary Information

Supplementary Figures.

Supplementary Video 1.

Supplementary Video 2.

## References

[1] Wang B, Facchetti A (2019). Mechanically flexible conductors for stretchable and wearable E-skin and E-textile devices. Adv. Mater..

[2] Lee Y (2020). Mimicking human and biological skins for multifunctional skin electronics. Adv. Funct. Mater..

[3] Sekitani T (2009). Stretchable active-matrix organic light-emitting diode display using printable elastic conductors. Nat. Mater..

[4] Chun KY (2010). Highly conductive, printable and stretchable composite films of carbon nanotubes and silver. Nat. Nanotechnol..

[5] Yamada T (2011). A stretchable carbon nanotube strain sensor for human-motion detection. Nat. Nanotechnol..

[6] Lee P (2012). Highly stretchable and highly conductive metal electrode by very long metal nanowire percolation network. Adv. Mater..

[7] Wang Y (2014). Wearable and highly sensitive graphene strain sensors for human motion monitoring. Adv. Funct. Mater..

[8] Amjadi M, Pichitpajongkit A, Lee S, Ryu S, Park I (2014). Highly stretchable and sensitive strain sensor based on silver nanowire-elastomer nanocomposite. ACS Nano.

[9] Helmer RJN (2011). A pilot evaluation of an electronic textile for lower limb monitoring and interactive biofeedback. Procedia Eng..

[10] Muth JT (2014). Embedded 3D printing of strain sensors within highly stretchable elastomers. Adv. Mater..

[11] Yao S, Zhu Y (2015). Nanomaterial-enabled stretchable conductors: strategies, materials and devices. Adv. Mater..

[12] Han JT (2017). Synthesis of nanobelt-like 1-dimensional silver/nanocarbon hybrid materials for flexible and wearable electroncs. Sci. Rep..

[13] Wang Y (2017). A highly stretchable, transparent, and conductive polymer. Sci. Adv..

[14] Matsuhisa N (2017). Printable elastic conductors by in situ formation of silver nanoparticles from silver flakes. Nat. Mater..

[15] Lei Z, Wu P (2019). A highly transparent and ultra-stretchable conductor with stable conductivity during large deformation. Nat. Commun..

[16] Azamian BR, Coleman KS, Davis JJ, Hanson N, Green MLH (2002). Directly observed covalent coupling of quantum dots to single-wall carbon nanotubes. Chem. Commun..

[17] Guo DJ, Li HL (2005). Highly dispersed Ag nanoparticles on functional MWNT surfaces for methanol oxidation in alkaline solution. Carbon.

[18] Zamudio A (2006). Efficient anchoring of silver nanoparticles on n-doped carbon nanotubes. Small.

[19] Kim YT (2006). Fine size control of platinum on carbon nanotubes: from single atoms to clusters. Angew. Chem. Int. Ed..

[20] Chen J, Lu G (2006). Controlled decoration of carbon nanotubes with nanoparticles. Nanotechnology.

[21] Ma PC, Tang BZ, Kim JK (2008). Effect of CNT decoration with silver nanoparticles on electrical conductivity of CNT-polymer composites. Carbon.

[22] Pasricha R, Gupta S, Srivastava AK (2009). A facile and novel synthesis of Ag-graphene-based nanocomposites. Small.

[23] Kholmanov IN (2012). Nanostructured hybrid transparent conductive films with antibacterial properties. ACS Nano.

[24] Hwang J (2013). Enhanced mechanical properties of graphene/copper nanocomposites using a molecular-level mixing process. Adv. Mater..

[25] Zhou Y (2013). Highly stable and dispersive silver nanoparticle-graphene composites by a simple and low-energy-consuming approach and their antimicrobial activity. Small.

[26] Han JT (2019). Structural recovery of highly oxidized single-walled carbon nanotubes fabricated by kneading and electrochemical applications. Chem. Mater..

[27] Ziegler KJ (2005). Controlled oxidative cutting of single-walled carbon nanotubes. J. Am. Chem. Soc..

[28] Price BK, Lomeda JR, Tour JM (2009). Aggressively oxidized ultra-short single-walled carbon nanotubes having oxidized sidewalls. Chem. Mater..

[29] Kim JD, Yun H, Kim GC, Lee CW, Choi HC (2013). Antibacterial activity and reusability of CNT–Ag and GO–Ag nanocomposites. Appl. Surf. Sci..

[30] Dresselhaus MS, Dresselhaus G, Saito R, Jorio A (2005). Raman spectroscopy of carbon nanotubes. Phys. Rep..

[31] Matsuhisa N (2015). Printable elastic conductors with a high conductivity for electronic textile applications. Nat. Commun..

[32] Kim TG (2018). Enhanced oxidation-resistant Cu@Ni core–shell nanoparticles for printed flexible electrodes. ACS Appl. Mater. Interfaces.

[33] Kim I (2018). A photonic sintering derived Ag flake/nanoparticle-based highly sensitive stretchable strain sensor for human motion monitoring. Nanoscale.

[34] Hwang BU (2015). Transparent stretchable self-powered patchable sensor platform with ultrasensitive recognition of human activities. ACS Nano.

[35] Gong S (2015). Tattoolike polyaniline microparticle-doped gold nanowire patches as highly durable wearable sensors. ACS Appl. Mater. Interfaces.

