# Supplementary material for: Synthesis of silver nanoparticles embedded with single-walled carbon nanotubes for printable elastic electrodes and sensors with high stability
Source: Sci Rep. 2021 Mar 4;11:5140. doi: 10.1038/s41598-021-84386-4 (PMC7933232; doi:10.1038/s41598-021-84386-4)
Supplement: Supplementary file 1 — Supplementary Figures. [file 41598_2021_84386_MOESM1_ESM.docx]

**Supplementary Information**

Synthesis of Silver Nanoparticles Embedded with Single-walled Carbon Nanotubes for Printable Elastic Electrodes and Sensors with High Stability

Jae Won Lee^1,2,⸸^, Joon Young Cho^1,3,⸸^, Mi Jeong Kim^4^, Jung Hoon Kim^1^, Jong Hwan Park^1^, Seung Yol Jeong^1^, Seon Hee Seo^1^, Geon-Woong Lee^1^, Hee Jin Jeong^1,*^, Joong Tark Han^1,3,*^

^1^Nano Hybrid Technology Research Center, Creative and Fundamental Research Division, Korea Electrotechnology Research Institute (KERI), Changwon 51543, South Korea

^2^Department of Physics, Pusan National University, Busan 46241, South Korea

^3^Department of Electro-Functionality Material Engineering, University of Science and Technology (UST), Changwon 51543, South Korea

^4^Department of Chemical Engineering, Pohang University of Science and Technology, Pohang 37666, South Korea

*Corresponding author.

E-mail addresses: [wavicle11@keri.re.kr](mailto:wavicle11@keri.re.kr) (H. J. Jeong).

E-mail addresses: [jthan@keri.re.kr](mailto:jthan@keri.re.kr) (J. T. Han).

^⸸^The authors contributed equally to this work.


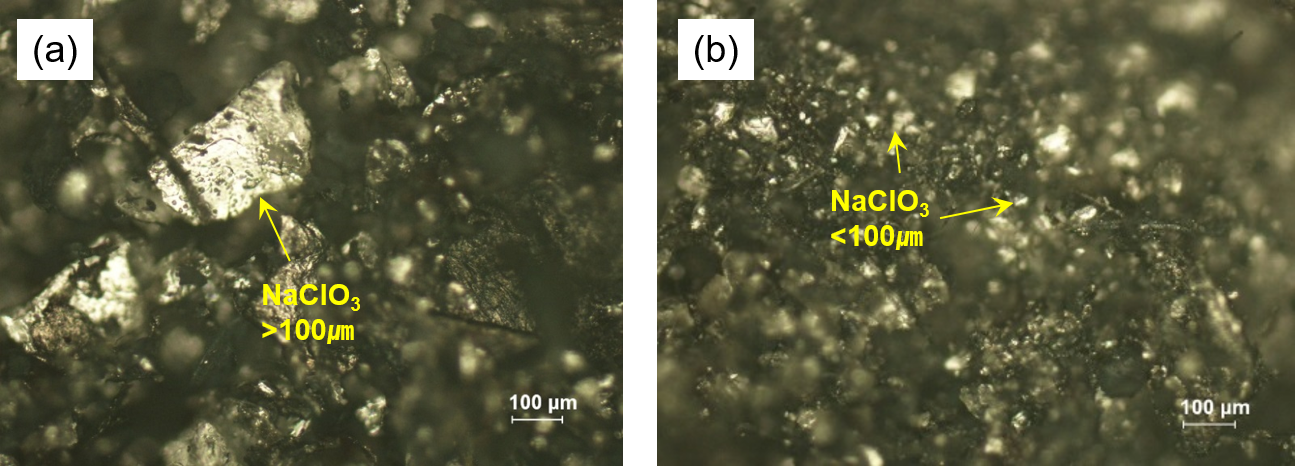


**Figure S1.** Optical images of SWCNT/NaClO_3_ mixtures after (a) mortar mixing and (b) high speed blade mixing.


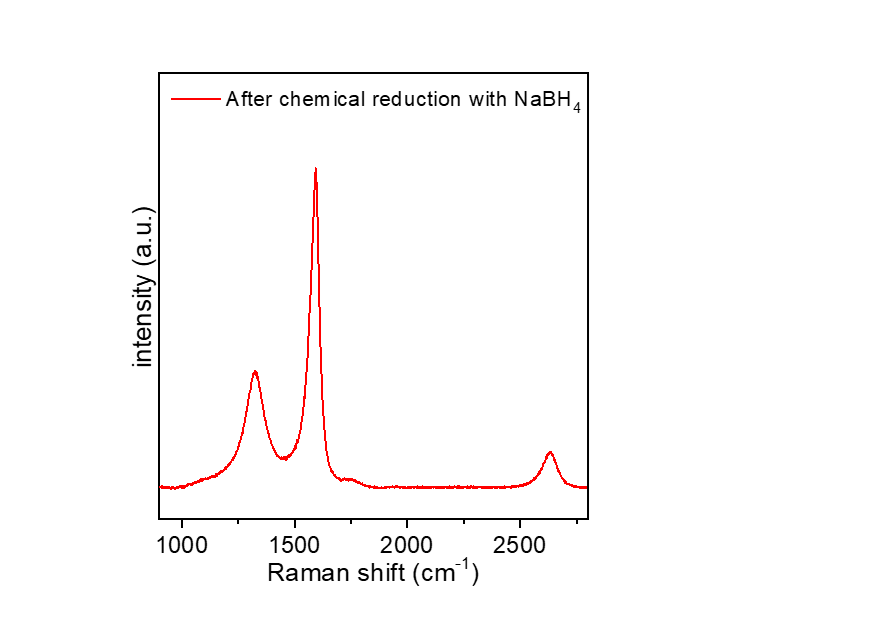


**Figure S2.** Raman spectrum of chemically reduced Ox-SWCNT with NaBH_4_.

**
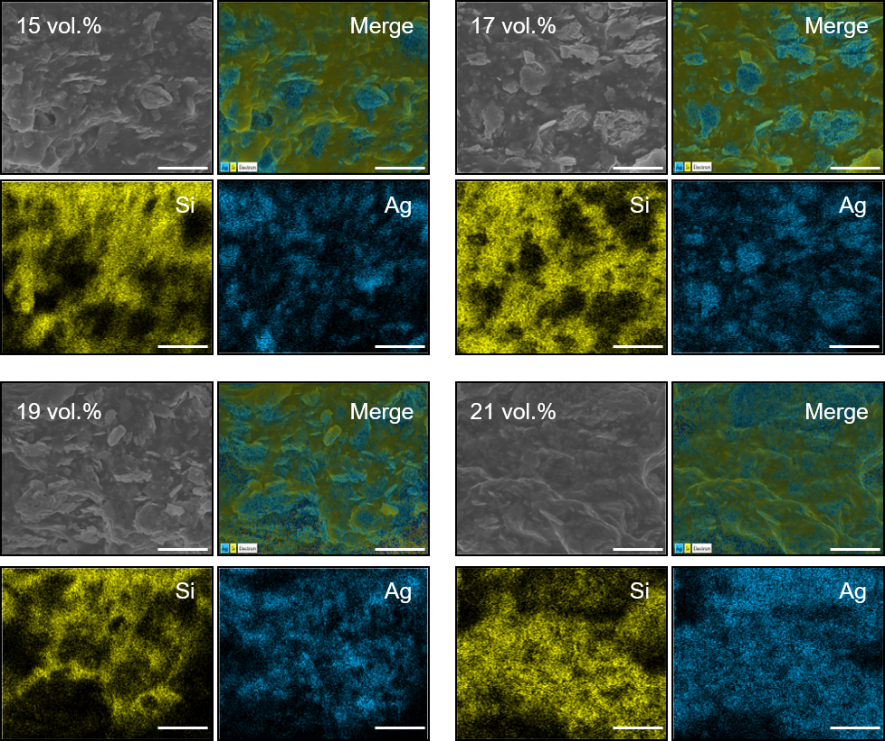
**

**Figure S3.** Cross-sectional SEM images of the elastic composite films with different filler contents from 15 to 21 vol.% after sintering at 18 J/cm^2^. The uniformity and density of the AgNPs/Ag flake conducting fillers was increased as the filler contents was increased. Especially, above the percolation threshold (19 and 21 vol.%), the conducting fillers are well distributed over entire area of composite film, resulting in higher electrical conductivity and better stretchability than low filler contents (15 and 17 vol.%) upon mechanical strain.


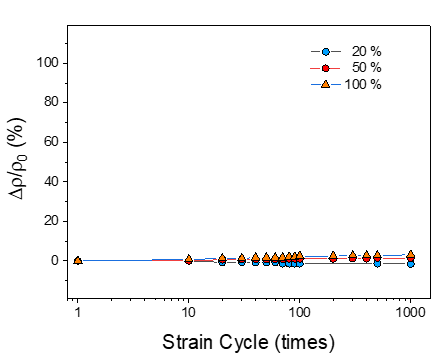


**Figure S4.** Repeatability test of up to 1,000 cycles for the composite film under 20, 50, and 100 % mechanical strains.

**
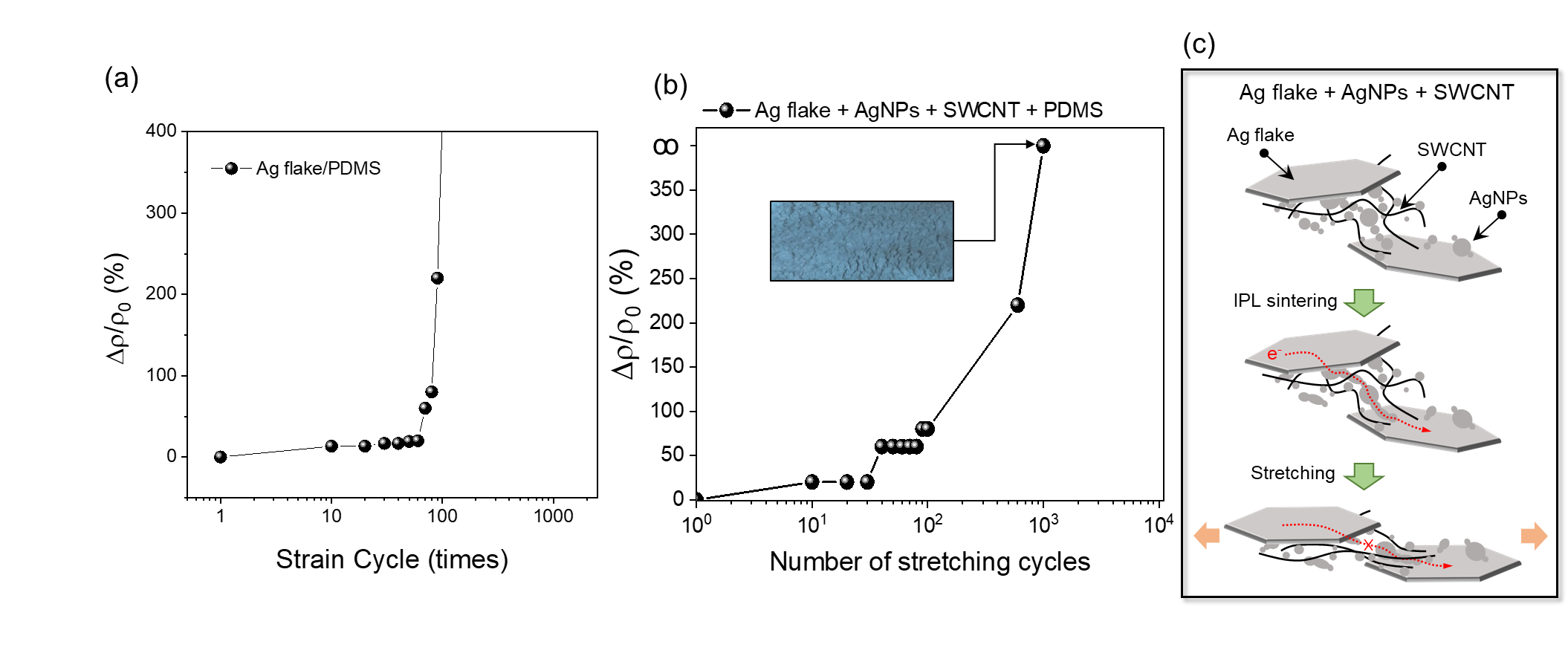
**

**Figure S5.** Repeatability test for the composite film composed of individually of (a) Ag flake and (b) Ag flake, AgNPs, and SWCNT. (c) Schematic image of electrical failure upon stretching.


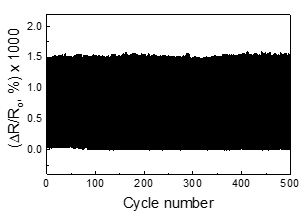


**Figure S6.** Repeatability test of up to 500 cycles for strain sensor of the composite film under 20 % mechanical strain.

**
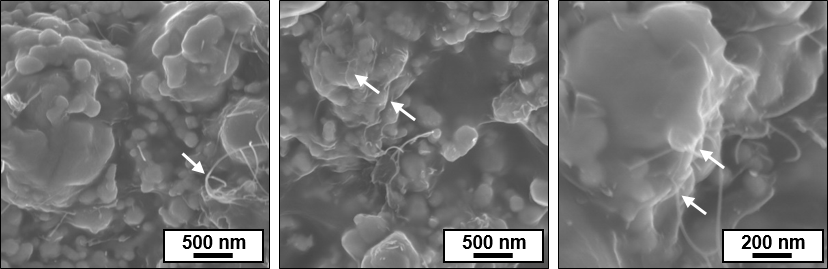
**

**Figure S7.** Cross-sectional FE-SEM images of the elastic composite films. The morphological structure of the SWCNT-embedded AgNPs was not damaged during the mixing of conductive fillers and PDMS. White arrows indicate the SWCNTs protruded from AgNPs.
